# Genetic profiles and hematological characteristics of the α-Hb variant Hb Chumphae (HBA2:c.32T>A) in association with –^SEA^ deletion α^0^-thalassemia

**DOI:** 10.1016/j.htct.2026.106510

**Published:** 2026-07-29

**Authors:** Patcharin Sihathip, Wibhasiri Srisuwan, Thitima Sumphanapai, Surada Satthakarn, Tabtim Ngokwongs, Sitthichai Panyasai

**Affiliations:** a Thalassemia Center, Khonkaen Hospital, Khonkaen, Thailand; b Department of Medical Technology, School of Allied Health Sciences, University of Phayao, Phayao, Thailand; c Faculty of Allied Health Sciences, Burapha University, Chonburi, Thailand; d Department of Medical Technology, Samsung Hospital, Khonkaen, Thailand

**Keywords:** Thalassemia, Hemoglobinopathies, Anemia, Hb Chumphae, α-globin variant

## Abstract

**Objective:**

Hemoglobin (Hb) variants and thalassemia interactions create complex syndromes, complicating diagnosis. This study aimed to characterize the molecular basis, genetic profile, and potential hematological features of a rare α-hemoglobin variant.

**Methods:**

A 44-year-old Thai male presented with unspecified chronic anemia with hepatosplenomegaly. Hematological data were obtained using a standard automated cell counter. Hemoglobin analysis was performed using high-performance liquid chromatography and capillary electrophoresis. Mutational and globin haplotypes were analyzed using polymerase chain reaction and sequencing. The pathogenicity of the mutant gene was predicted, and novel diagnostic methods based on allele-specific polymerase chain reaction were developed.

**Results:**

Hematological analysis revealed the following: red blood cell count, 1.74 × 10¹²/L; hemoglobin, 3.7 g/dL; hematocrit, 17%; mean corpuscular volume, 84.7 fL; mean corpuscular hemoglobin, 21.3 pg; mean corpuscular hemoglobin concentration, 25.1 g/dL; and red cell distribution width, 40.5%. Further, Hb A_2_ABart’s with abnormal peaks migrated faster than Hb A. DNA sequencing identified a missense mutation at codon 10 (GTC>GAC), causing a valine-to-aspartic acid substitution responsible for Hb Chumphae. Additionally, an α^0^-thalassemia (SEA deletion) was identified. Predictions and classifications of pathogenicity indicated a pathogenic effect. Polymerase chain reaction-restriction fragment length polymorphisms and allele-specific polymerase chain reaction assays successfully identified this mutation. An α-haplotypic [− − M − − − −] was possibly associated with Hb Chumphae.

**Conclusions:**

The presence of Hb Chumphae in combination with α^0^-thalassemia as a compound heterozygote leads to severe anemia worse than hemoglobin H disease. The diagnosis of Hb Chumphae is challenging as high-performance liquid chromatography and capillary electrophoresis chromatograms mimic those of Hb Bart’s. Accurate diagnosis requires globin genotyping.

## Introduction

Alpha-thalassemia is a common inherited hemoglobin (Hb) disorder in Southeast Asia, particularly in Thailand, where both deletional and non-deletional mutations contribute to a wide spectrum of clinical phenotypes [1]. Hb H disease arises from the loss or dysfunction of three of four *α-globin* genes, typically due to the compound heterozygosity of α^0^-thalassemia (--) and α^+^-thalassemia (-α or α^mutation^α) [2]. While most α^0^-thalassemia cases in Thailand result from --^SEA^ and --^THAI^ deletions, rare deletional forms involving the upstream α-major regulatory element (α-MRE) that render both *α-globin* genes non-functional (αα)^T^ should also be considered in molecular diagnosis. The clinical severity of Hb H disease ranges from moderate to severe anemia, depending on the nature of the *α-globin* gene mutations. Patients with non-deletional forms often present with more severe symptoms than those with deletional forms. [3].

To date, approximately 840 human Hb variants resulting from *α-globin* gene mutations have been identified worldwide. Although most α-Hb variants are clinically silent, some can cause significant hematological abnormalities. Clinically severe phenotypes arise when these variants co-occur with α^0^-thalassemia, classified as non-deletional Hb H disease [4]. In Thailand, over 60 Hb variants have been reported, with a prevalence of approximately 2.4% [[5], [6], [7]]. The most frequently observed α-Hb variants include Hb Constant Spring, Hb Paksé, and Hb Q-Thailand (HBA1:c.223G>C), often found in combination with α⁺-thalassemia (-α^4.2^ kb) deletions [6]. Other notable variants include Hb Quong Sze (HBA2:c.377T>C), Hb Suan Dok (HBA2:c.329T>G), Hb Pak Num Po (HBA1:c.396_397insT), and Hb Adana (HBA2 or HBA1:c.179G>A). Importantly, these variants are often associated with thalassemia, causing various clinical phenotypes [1,8,9].

Interactions between these Hb variants and α-thalassemia mutations can result in complex syndromes, complicating diagnosis and genetic counseling. A novel α-globin variant, Hb Chumphae (HBA2:c.32T>A, α2 10(A8) Val→Asp), was recently identified in a Thai newborn with compound heterozygosity for Hb Chumphae and the Southeast Asian α^0^-thalassemia deletion (--^SEA^). It was undetectable by standard Hb electrophoresis in heterozygous carriers. This combination resulted in a previously undescribed form of Hb H disease, characterized by moderate anemia, microcytosis, and comorbid Hb Bart’s and Hb H, indicating a more severe phenotype than classical deletional Hb H disease [10]. The complete molecular basis of the thalassemic phenotype associated with this variant remains to be elucidated, particularly the mechanisms governing its clinical expression.

This study aimed to investigate the genetic profiles and hematological characteristics of Hb Chumphae in a Thai patient with Hb H disease and understand its pathophysiology.

## Materials and methods

### Patient, hematological analysis, and identification of Hb variants

This study was reviewed and approved by the Institutional Review Board of the University of Phayao, Phayao, Thailand (1.2/026/67) and was conducted according to the tenets of the Declaration of Helsinki. Written informed consent was obtained from the patient.

The patient was a 44-year-old Thai male who presented with a three-week history of tachypnea, progressive dyspnea on exertion, non-productive cough, and low-grade fever. His past medical history was notable for chronic anemia of unknown etiology, for which he had received intermittent blood transfusions, the last being administered when the patient was 39 years old. Six months after this last transfusion, the patient underwent a complete blood count examination at a local hospital, which revealed: red blood count (RBC), 3.99 × 10¹²/L; Hb, 9.0 g/dL; hematocrit (Hct), 31.9%; mean corpuscular volume (MCV), 79.9 fL; mean corpuscular hemoglobin (MCH), 22.6 pg; mean corpuscular hemoglobin concentration (MCHC), 28.2 g/dL, and red cell distribution width (RDW), 29.9%. These values, obtained from the medical records, represent the patient's steady-state hematological profile in the absence of acute illness and at least six months after any transfusion. Physical examination and abdominal ultrasonography indicated hepatosplenomegaly. He was diagnosed with congestive heart failure secondary to chronic anemia of unknown etiology, complicated by pneumonia. A peripheral blood sample anticoagulated with ethylenediaminetetraacetic acid was collected prior to blood transfusion for further hematological and molecular analysis. Blood specimens and initial hematological data from the hospital were sent to the School of Allied Health Sciences, University of Phayao. Hb analysis was performed using two automated cation-exchange high-performance liquid chromatography (HPLC) systems: VARIANT II (Bio-Rad, Hercules, CA, USA) and β-Thalassemia Short Program and Premier Resolution (Trinity Biotech, in Bray, County Wicklow, Ireland). The latter is a novel cation exchange chromatography quantifying Hb A_2_ even in the presence of Hb E in high-resolution mode. Capillary electrophoresis (CE) was performed using the MINICAP Flex Piercing system (Sebia, Lisses, France).

### Molecular analysis

Genomic DNA was extracted from peripheral blood leukocytes using the Total Blood DNA Isolation Kit (Vivantis Technologies, Selangor, Malaysia). Common α-thalassemia mutations (–^SEA^, –^THAI^, -α^3.7^, -α^4.2^, Hb Constant Spring (HBA2:c.427T>C) [α^CS^], and Hb Paksé (HBA2:c.429A>T) [α^Paksé^]) were identified using gap-polymerase chain reaction (PCR) and allele-specific PCR (AS-PCR) [[11], [12], [13]]. AS-PCR was also used to identify Hb variants with the same retention time (RT) on Hb-HPLC and migration zones on the electropherogram as those occasionally documented in the Thai population. These variants included Hb Hekinan II (HBA1:c.84G>T) and Hb Wiangpapao (HBA1:c.133C>T) [14]. Mutational analyses of *α1*-, *α2*-, and *β*-globin genes were performed by amplifying their sequences using a previously described protocol [15,16]. Direct DNA sequencing of amplified globin genes was performed using an ABI PRISM™ 3130 XL analyzer (Applied Biosystems, Foster City, CA, USA).

Mutation positions were identified and analyzed using the human abnormal Hb thalassemia libraries HbVar (http://globin.bx.psu.edu/hbvar) and IthaGenes (https://www.ithanet.eu/db/ithagenes). Variants were named according to HbVar (http://globin.bx.psu.edu/cgi-bin/hbvar/query_vars3). The *α-globin* gene haplotype was assessed using PCR-based restriction enzyme digestion at one broadly triallelic inter-ζ-globin hypervariable region (HVR) and at six polymorphic restriction sites: (1) the *Xba*I site of the 5′ *ζ2*-globin gene, (2) *Sac*I site of the 3′ *ζ2*-globin gene, (3) *Acc*I site of the 3′ *ψα2*-globin gene, (4) *Rsa*I site of the 5′ *α2*-globin gene, and (5) *Pst*I sites of the 5′ *α1*-globin gene and (6) 5′ *θ1*-globin gene. The PCR products containing each of these polymorphic sites were amplified via PCR and digested with appropriate restriction enzymes [17]. Haplotypes were determined by analyzing the presence or absence of cleavage at each site and combining these outcomes into a unified pattern.

### The development of polymerase chain reaction-restriction-fragment length polymorphism to identify HBA2:C.32T>A (Hb Chumphae)

The GTC–GAC mutation in codon 10 of the *α2-globin* gene, causing Hb Chumphae, abolishes a BstPAI recognition site (5′-GACNN^▼^NNGTC-3′). PCR-restriction fragment length polymorphism (RFLP) assays were developed to detect this mutation. A 1085-base pair (bp) *α-globin* gene fragment was amplified using primers C1 (5′-TGGAGGGTGGAGACGTCCTG-3′) and C3 (5′-CCATTGTTGGCACATTCCGG-3′) under specific PCR conditions [15]. PCR amplification was performed in a total volume of 50 µL containing 100–200 ng genomic DNA, 60 pmol of each primer, and 1.5 units of *Taq* DNA polymerase (Vivantis) in 10 mM Tris–HCl (pH 9.1), 50 mM KCl, and 0.1% Triton™ X-100. For restriction digestion, the 10 µL of amplified fragment was completely digested by 1 unit of BstPAI (New England Biolabs, Beverly, MA, USA) at 37 °C for six hours. Digested fragments were then analyzed using 1.5% agarose gel electrophoresis and visualized under ultraviolet (UV) light after ethidium bromide staining. The 1085-bp amplified fragment from the normal allele (α^A^) was digested into 818-bp and 267-bp fragments, whereas the Hb Chumphae allele (α^Chumphae^) remained undigested.

### Allele-specific polymerase chain reaction to identify Hb Chumphae

A simple rapid DNA assay using AS-PCR was developed for the GTC–GAC mutation in Hb Chumphae. The forward primer SP60 (5′-CCTGCCGACAAGACCAACGA-3′) and common primer C3 produced an 834-bp fragment specific to the α^Chumphae^ allele. Two additional primers, γ4 (5′-GGCCTAAAACCACAGAGAGT-3′) and γ5 (5′-CCAGAAGCGAGTGTGTGGAA-3′), generated a 578-bp internal control fragment. The total PCR mixture (50 μL) contained 100–200 ng genomic DNA, 24 pmol SP60 and C3, 60 pmol γ4 and γ5, 200 μM dNTPs, and 2.0 units *Taq* DNA polymerase (Vivantis) in 10 mM Tris–HCl (pH 9.1), 50 mM KCl, and 0.1% Triton™X-100. Amplification was performed using a thermal cycler (Cycler Personalis, Bio-Rad, USA). After an initial denaturation step at 94 °C for 3 min, the reaction was followed by 30 cycles at 94 °C for 30 s, 64 °C for 30 s, 72 °C for 1 min, and a final extension at 72 °C for 10 min. The amplified products were analyzed via 1.5% agarose gel electrophoresis and visualized under UV light after ethidium bromide staining.

### Variant classification and bioinformatics analysis of Hb Chumphae

Variant pathogenicity was evaluated using the American College of Medical Genetics and Genomics and Association for Molecular Pathology (ACMG/AMP) best-practice guidelines, as specified by the ClinGen Hemoglobinopathy Variant Curation Expert Panel [18,19]. Additionally, the impact of the amino acid substitution on the *α-globin* chain and the structure and function of the *α-globin* chain were predicted using network-based HumDiv-trained Polymorphic Phenotype V.2 (PolyPhen-2) (http://genetics.bwh.harvard.edu/pph2/) and the Sorting Intolerant from Tolerant (SIFT) web server (https://sift.bii.a-star.edu.sg/) [20]. Additionally, the PredictSNP program, freely available at https://loschmidt.chemi.muni.cz/predictsnp1/, was used to estimate whether the effect of amino acid substitution was neutral or deleterious [21]. In addition, homology models of three-dimensional (3D) structures of wild-type and variant proteins were generated from amino acid sequences using the SWISS-MODEL website [22,23]. The crystal structure of deoxygenated Hb complexed with 5HMF-NO (PDB code: 7ud7.1) was selected based on sequence identity. The resulting 3D protein structures were visualized using Discovery Studio Visualizer and PyMOL (Biovia, D. S., Discovery Studio Visualizer, v24.1.0.23298; Dassault Systems, San Diego, CA, 2024, and Schrodinger, LLC, The PyMOL Molecular Graphics System, Version 3). Swiss-PdbViewer version 4.1 was used to predict contact sites of residues of interest [24]. The crystal structure of alpha Hb-stabilizing protein (α-HSP; PDB code 1Y01) was used as a template for α-HSP and *α-globin* chain binding simulation. To investigate the interaction between α-HSP and *α-globin* chain in either native or mutant HBA2 proteins, protein–protein docking simulation was performed using ZDOCK (ZDOCK 3.0.2, https://zdock.umassmed.edu). Contracting residues for docking were selected based on α-HSP interaction sites with the *α-globin* chain.

## Results

### Hematological and Hb findings

**Figure 1:**
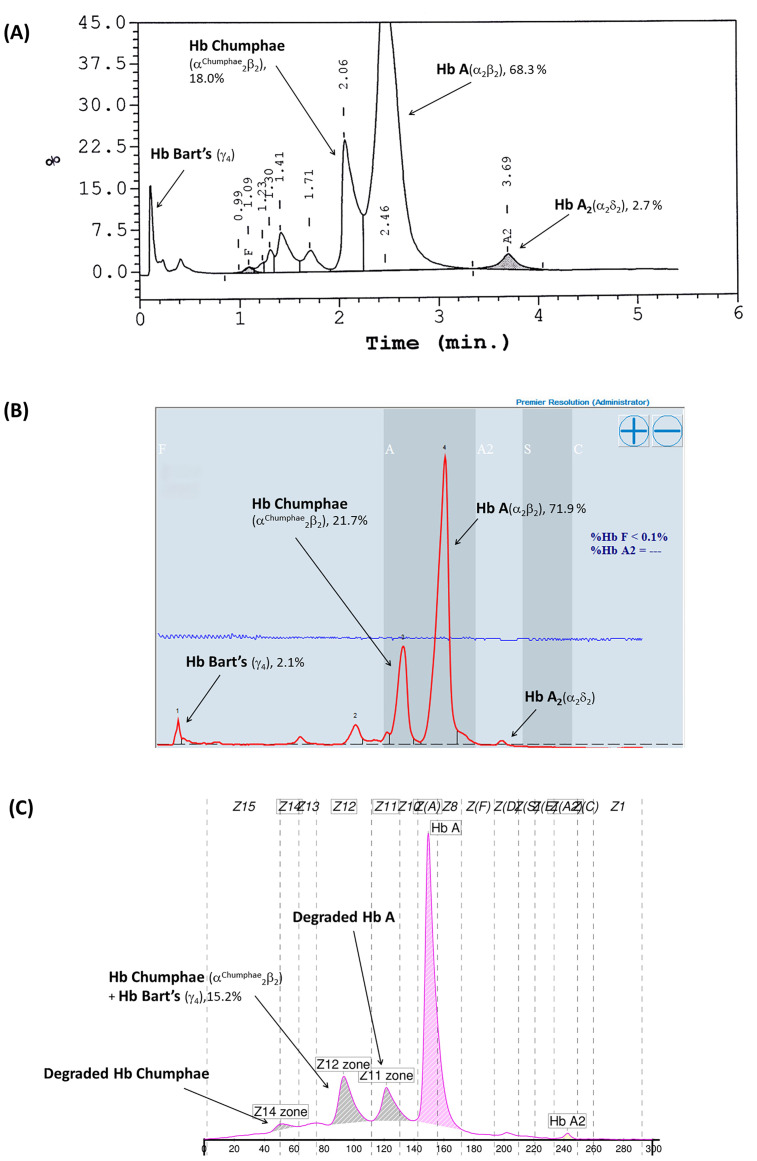
Hemoglobin (Hb) analysis results obtained using three different automated Hb analyzers. **(A)** Hb-chromatogram profile on the HPLC-Variant II system. **(B)** Hb-chromatogram profile on the HPLC-Premier Resolution system. **(C)** Hb-electropherogram pattern by capillary electrophoresis.

The patient’s hematological findings indicated severe anemia: (RBC count: 1.74 × 10^12^/L, Hb: 3.7 g/dL; Hct: 17.0%; MCV: 84.7 pg; MCH: 21.3 pg; MCHC: 25.1 g/dL; and coefficient of variation of RDW: 40.5%; reticulocyte count: 7.7%). The serum ferritin level during the acute crisis was elevated at 930.3 ng/mL (reference range: 23.9–336.2 ng/mL). A blood smear showed hypochromia and anisocytosis. The VARIANT II HPLC revealed abnormal Hb peaks eluting faster than Hb A peaks. This abnormal Hb peak was incompletely eluted from Hb A at a specific RT of 2.06 min, accounting for 18.0% of the total Hb (Figure 1A). Additionally, Hb Bart’s (γ_4_) was distinctly observed on the chromatogram. A comparable analysis using the Premier Resolution-HPLC system detected an abnormal Hb peak completely eluting from Hb A at RT of 3.882 min (21.7% of the total Hb) and Hb H and Hb Bart's at RT of 0.330 min (2.1% of the total Hb) (Figure 1B). Similarly, CE Hb analysis showed an abnormal Hb peak in migrating zone 12, resembling Hb Bart’s, and accounting for 15.2% of the total Hb (Figure 1C). A comprehensive overview of Hb analysis profiles and levels is presented in Table 1.

**Table 1:** Hematological data, red blood cell indices, and *α-globin* genotypes of the patient.

Parameter	Steady-State^a^	Present^b^	Reference range
Red blood cell parameters			
RBC count (x 10^12^/L)	3.99	1.74	3.8–5.5
Hb (g/dL)	9.0	3.7	12.0–16.0
Hct (%)	31.9	17.0	36.0–46.0
MCV (fL)	79.9	84.7	80.0–97.0
MCH (pg)	22.6	21.3	27.0–31.0
MCHC (g/dL)	28.2	25.1	32.0–36.0
RDW-CV (%)	29.9	40.5	11.0–15.0
Reticulocyte count (%)	na	7.7	0.2–2.0
Serum ferritin (ng/mL)	na	930.3	23.9–336.2
Hemoglobin analysis			
HPLC—Hb Profile^c^	na	A_2_ABart's with Hb Chumphae	
Hb A (%)		68.3	>95.0
Hb A_2_ (%)		2.7	2.0–3.5
Hb Chumphae (%)		18.0	not
Hb F (%)		0.6	<1.0
HPLC—Hb Profile^d^	na	A_2_ABart's with Hb Chumphae	
Hb A (%)		71.9	>95.0
Hb A_2_ (%)		nd	2.0–3.5
Hb Chumphae (%)		21.7	not
Hb F (%)		<0.1	<1.0
Hb Barts (%)		2.1	
CE-Hb Profile^e^	na	A_2_A with Hb Chumphae	
Hb A (%)		71.9	>95.0
Hb A_2_ (%)		0.9	2.0–3.5
Hb Chumphae + Hb Barts (%)		15.2	not
α-globin genotype		--^SEA^/α^10T>A^α	
α-globin haplotype		[±, −, S/M, −, −, −, −]	−

RBC: red blood cell; Hb: hemoglobin; Hct: hematocrit; MCV: mean corpuscular volume; MCH: mean corpuscular hemoglobin; MCHC: mean corpuscular hemoglobin concentration; RDW-CV: coefficient of variation of the red cell distribution width; na: not available.

ᵃSteady-state parameters obtained six months after the last blood transfusion (patient age 39 years).

ᵇCrisis-state parameters obtained during acute hemolytic crisis presentation (patient age 44 years), prior to transfusion.

c Determined using high-performance liquid chromatography, β-THAL short program, Bio-Rad Variant II system.

d Determined using high-performance liquid chromatography, Premier Resolution system.

e Determined using capillary electrophoresis, MINICAP Flex Piercing system.

### Globin gene abnormalities

**Figure 2:**
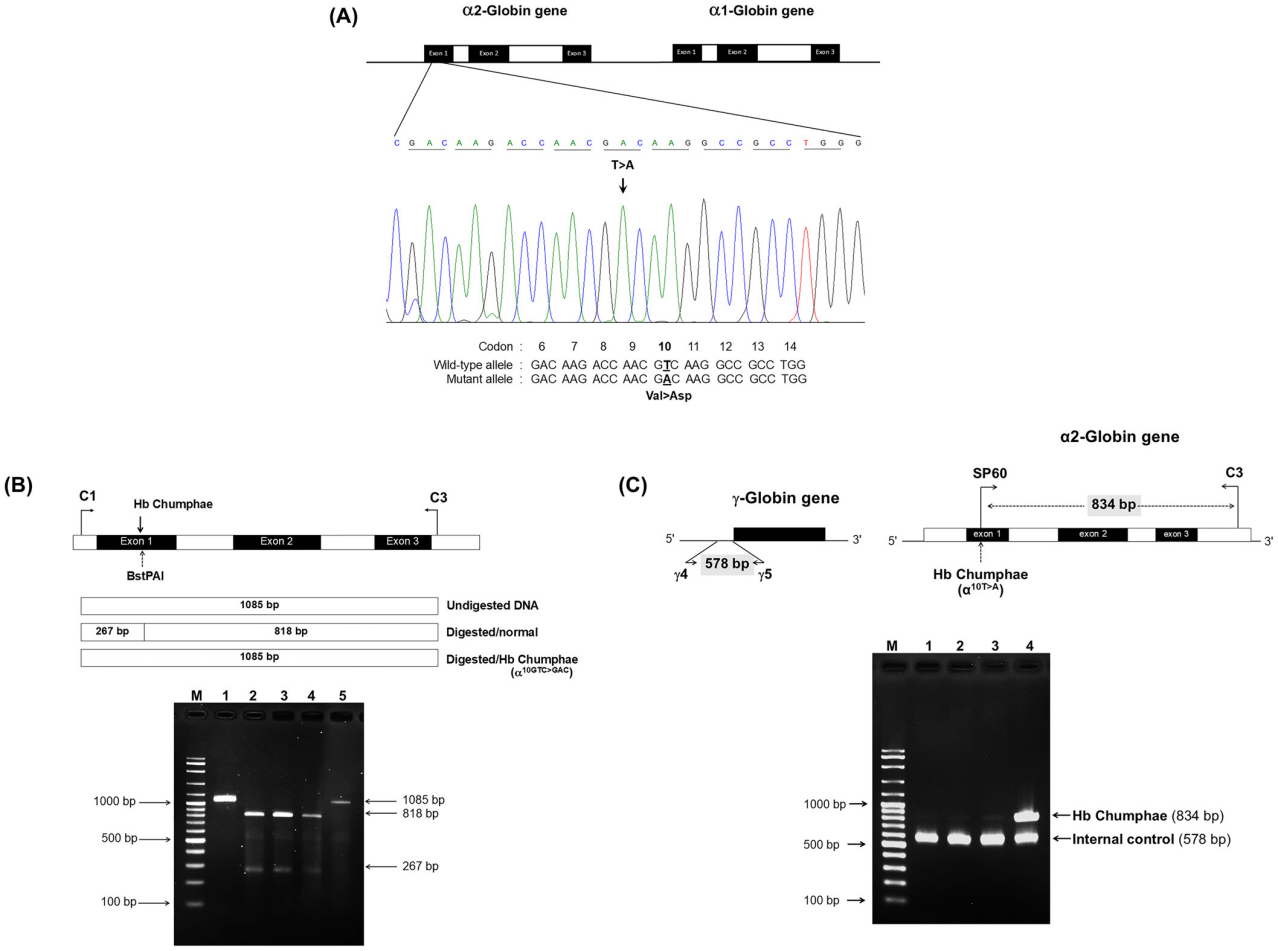
Mutation identification using DNA sequencing, polymerase chain reaction (PCR)-restriction fragment length polymorphism, and allele-specific PCR. **(A)** The sequencing profile of the *α2-globin* gene shows antisense strand sequences in codon 10, where the GTC–GAC mutation causes hemoglobin (Hb) Chumphae. **(B)** GTC-GAC mutation identification using BstPAI digestion of the PCR product. M represents the 100-bp DNA ladder markers. Lane 1 displays undigested amplified DNA for gel electrophoresis. Lanes 2–5 show digested amplified DNA samples. Lanes 2–4 contain BstPAI-digested amplified DNA from non-mutated samples, showing 818- and 267-base pair (bp) digested fragments. Specifically, lane 4 also has a 1085-bp undigested fragment, indicating the presence of Hb Chumphae. **(C)** Hb Chumphae mutation is identified using an allele-specific PCR assay. Primers SP60 and C3 amplify an 834-bp fragment specific to Hb Chumphae, whereas primers γ4 and γ5 amplify a 578-bp internal control band. M represents the 100-bp DNA ladder markers. Lane 1: negative DNA control; lanes 2 and 3: normal DNA samples; lane 4: DNA of the patient with Hb Chumphae co-inherited with α^0^-thalassemia.

Sequence analysis of the selectively amplified *α-* and *β-globin* genes revealed a pattern consistent with homozygosity for a missense mutation at codon 10 (GTC>GAC) in the *α2-globin* gene. This mutation caused the valine-to-aspartic acid substitution [α10(A8)Val>Asp; HBA2:c.32T>A] (Figure 2A) responsible for Hb Chumphae. Notably, only A nucleotide was identified at this position, indicating that one chromosome carried the *α^0^-thalassemia* gene with two deleted *α-globin* genes. Consequently, only one *α2-globin* gene was amplified for DNA sequencing. Further DNA analysis confirmed SEA deletion α^0^-thalassemia, indicating that the patient coinherited Hb Chumphae with α^0^-thalassemia, corresponding to the genotype (--^SEA^/α^10T>A^α). No mutations were detected in the *β-globin* gene.

### Mutated Hb variants identified by polymerase chain reaction-restriction fragment length polymorphism

The GTC to GAC mutation at codon 10 abolished the BstPAI restriction site (5′-GACNN^▼^NNGTC-3′) on the *α-* gene. This mutation was confirmed using PCR–RFLP analysis (Figure 2B). In the normal allele, the 1085-bp fragment was digested into 267-bp and 818-bp fragments, whereas the fragment in the Hb Chumphae allele remained undigested. The developed PCR–RFLP method successfully confirmed the presence of Hb Chumphae in the patient.

### Mutated Hb variants identified by allele-specific polymerase chain reaction

A new AS-PCR technique was developed for rapid DNA diagnosis of Hb Chumphae. The amplified 834-bp fragment from the Hb Chumphae allele was clearly observed in patients harboring Hb Chumphae, whereas the 578-bp fragment specific for the normal allele was observed in normal individuals (Figure 2). This result indicated that the newly developed AS-PCR technique successfully enabled rapid diagnosis of Hb Chumphae.

### α-Globin gene haplotypes

Further *α-globin* gene haplotype analysis revealed a haplotype (±, −, S/M, −, −, −, −) associated with Hb Chumphae. Two polymorphic sites showed heterozygous patterns: the *Xba*I site of the 5′ *ζ2-globin* gene and the broadly triallelic inter-ζ-globin HVR. These two polymorphic sites are commonly found in the α^A^ allele [25,26]. Accurate segregation analysis was not possible owing to a lack of specimens from family members. However, the α^Chumphae^ allele was possibly associated with haplotype (−, −, M, −, −, −, −), whereas the α^A^ allele was associated with haplotype (+, −, S, −, −, −, −).

### Molecular model and pathogenicity

**Figure 3:**
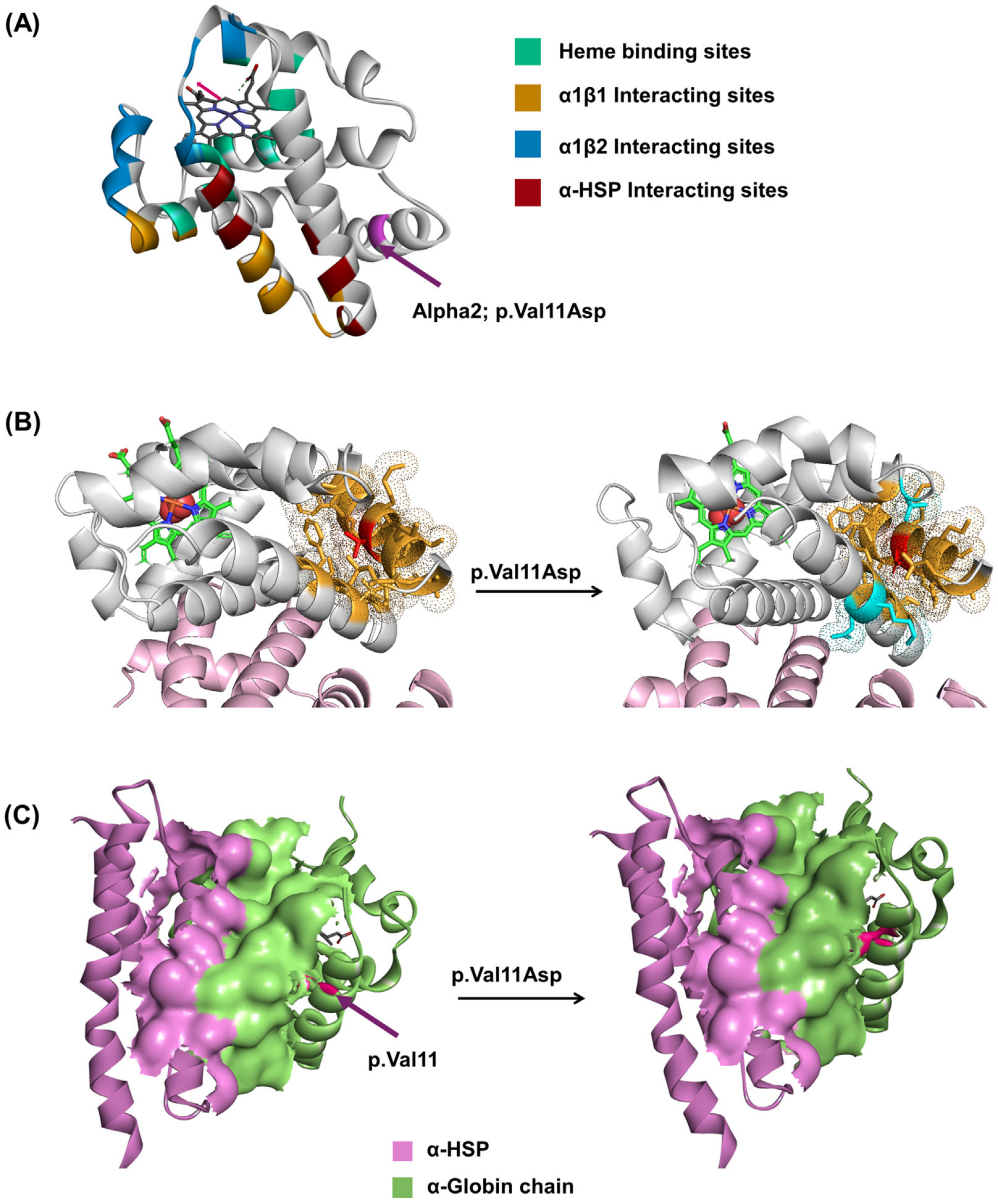
Homology modeling of HBA2 proteins demonstrating the α-globin chain in a ribbon structure. Methionine is numbered as 1 in this format, regardless of processing. **(A)** In the three-dimensional (3D) structure of HBA2 proteins, residues are labeled according to their binding sites, and mutation position is labeled in violet. **(B)** In the 3D structure of hemoglobin, the *α-globin* chain is gray, and the *β-globin* chain is pink. The normal α-chain is on the left, and the mutated α-chain is on the right. Side chains of residue position 11 are red, side chains of residues that interact with residue position 11 are brown, and side chains of additional interacting residues with mutant position 11 are blue. **(C)** The 3D structures of α-HSP (purple) and α-globin chain (green) are shown in ribbon representation. Contracting areas of the protein complex are shown in surface representation. Residue position 11 is shown as pink.

The mutation site was located outside of the α1β1 or α1β2 interface and crucial functional binding sites for oxygen, proteins, and heme (Figure 3A). Interaction predictions showed that the residue at position 11 (valine in the normal α-chain on the left and aspartic acid in the mutated α-chain on the right) was involved in intra-chain interactions within the monomer (Figure 3B). The replacement of valine with aspartic acid resulted in significant changes in the interactions; aspartic acid carries a negative charge and likely disrupts the hydrophobic interactions originally stabilized by valine. The introduction of aspartic acid created new interactions, specifically with residues Val74, Asp127, and Lys128. These additional interactions stabilized or destabilized the Hb structure.

In protein–protein docking simulations, the top-ranked poses for each complex yielded ZDOCK scores of 1227.522 and 1253.109, respectively, suggesting favorable binding configurations. Valine to aspartic acid substitution at this position did not significantly affect intermolecular interactions (Figure 3C). Furthermore, pathogenicity predictions for Hb Chumphae showed an SIFT score of 0.00 and a PolyPhen-2 score of 0.999, classifying it as probably damaging. Furthermore, PredictSNP predicted the mutation as deleterious. Additionally, the HBA2:c.32T>A mutation met the ACMG/AMP criteria to be classified as ‘pathogenic’ for α-thalassemia and related disorders (Table 2).

**Table 2:** American College of Medical Genetics and Genomics and Association for Molecular Pathology (ACMG/AMP) criteria for pathogenicity classification of the HBA2:c.32T>A variant including in silico predictions.

**Criterion**	**Evidence**
PVS1	Null allele: Amino acid transition of valine to aspartic acid disturbed initial step in protein folding
PP3	Three silico predictive programs have consistent predictions. SIFT, PolyPhen 2, and PredictSNP predict that the HBA2:c.32T>A mutation has deleterious effects.
PM2_P	Absent from the gnomAD v4.1.0 database
PM3	Found in trans with SEA deletion α^0^-thalassemia

## Discussion

Hb Chumphae was identified in a patient with compound heterozygosity for Hb Chumphae and α^0^-thalassemia associated with severe anemia, abnormal Hb migration patterns, and undetectable levels of Hb H, despite the expected synthesis. Detailed insights into the physicochemical and electro-mobility behavior of this Hb variant will enhance our understanding of its role in thalassemia syndromes and improve diagnostic protocols for affected individuals.

Hb Chumphae is an *α-globin* chain variant characterized by a GTC>GAC mutation at codon 10 of the *α2-globin* gene, resulting in the substitution of valine with aspartic acid. This substitution introduces an additional negative charge at the α-chain side chain per molecule, altering the net charge of the Hb Chumphae tetramer. Consequently, Hb Chumphae (α^Chumphae^₂β₂) elutes earlier than Hb A (α₂β₂) on both HPLC devices, enabling its distinction from Hb A. Notably, the Hb Chumphae derivative A_2_-Chumphae, formed from the (α^Chumphae^_2_δ_2_) tetrameric assembly, was undetectable by HPLC and CE. It was possibly concealed within Hb A_2_ or synthesized in minimal quantities. Despite Hb Chumphae separation, it migrates to the Hb J zone (zone 12), with similar migration velocities to Hb Bart’s and regional Hb variants, such as Hb Prato, Hb J-Buda, Hb J-Singapore, Hb Shenyang, Hb Pyrgos, and Hb J-Bangkok [[27], [28], [29], [30]].

Additionally, its mobility pattern on the HPLC-VARIANT II system resembles that of Hb Hekinan (α27[B8]Glu>Asp), Hb Wiangpapao (α27[B8]Glu>Asp]), and Hb Murica [31,32]: the first two variants are occasionally found in the Thai population. Hb Hekinan (α27[B8]Glu>Asp), Hb Wiangpapao (α27[B8]Glu>Asp]), and Hb Murica are not completely separated from Hb A, complicating diagnosis when using the HPLC-VARIANT II system. Fortunately, the Premier Resolution HPLC system effectively differentiates Hb Chumphae from Hb A and Hb Bart’s, allowing for accurate quantification in cases where other methods are inconclusive. To the best of our knowledge, this is only the second reported case of Hb Chumphae in Thailand. These findings indicate that Hb Chumphae moves faster than Hb A and can be separated from Hb A using HPLC. However, it co-migrates with Hb Bart’s during CE. This observation contrasts with previous findings where the same genotype was undetectable in peripheral blood owing to high instability [10].

In the current study, the patient with the --^SEA^/α^Chumphae^α genotype showed low circulating Hb Chumphae levels in peripheral blood when estimated by different methods. This patient had only two *α-globin* genes: a mutated *α2* gene producing the α^Chumphae^ globin chain and an *α1* gene producing the α^A^ globin chain (arranged as 5ˈ-α2-α1–3ˈ in a haploid). Thus, Hb Chumphae concentrations were expected to be 2–3 times higher than Hb A concentrations. The average protein production rate of the *α2-globin* gene is 2–3 times higher than that of the *α1-globin* gene in normal individuals [33]. However, Hb Chumphae accounted for approximately 15.2%–21.7% of total Hb by HPLC. The α-globin variant expression within the RBC depends on several factors, including the *α-globin* locus type carrying the mutation, translation efficiency of mutant α-globin mRNA, protein subunit folding, tetramer assembly, and overall tetramer stability [34]. In silico analysis in the current study provided crucial insights on the probable mechanisms resulting in the low percentage of Hb Chumphae in peripheral blood. The results showed that the Hb Chumphae mutation site was located outside crucial α1β1 or α1β2 interfaces and binding sites for oxygen, proteins, and heme.

These findings were confirmed by the PyMOL program analysis, indicating that amino acid substitution at this position did not affect the structural stability or functional properties of the Hb molecule. However, the mutation may still influence protein stability and function. Homology modeling of the 3D structures of both wild-type and variant proteins via the SWISS-MODEL revealed significant structural alterations. Additionally, the crystal structure of deoxygenated Hb complexed with 5HMF-NO (PDB code: 7ud7.1) served as a template for modeling Hb Chumphae. The resulting models, visualized using Discovery Studio Visualizer and PyMOL, showed structural changes induced by the Val-to-Asp substitution. Particularly, an additional negative charge was introduced, altering the net charge of the Hb Chumphae tetramer. PolyPhen-2, SIFT, and PredictSNP corroborated these findings, suggesting that the amino acid substitution had a deleterious effect.

Despite the observation that the valine residue at position 11 forms three additional intra-chain interactions relative to Hb A, the mutation exerts no impact on inter-chain stability. Additionally, ZDOCK simulations showed that the amino acid changes did not affect the binding interaction between α-HSP and the mutant α-globin. α-HSP binds to α-Hb, inhibiting the generation of α-Hb-reactive oxygen species and their precipitation under oxidant stress [35]. The current analysis found that the α^Chumphae^ chain is still stabilized by α-HSP, preventing rapid chain proteolysis or precipitation. The results indicate that low Hb Chumphae levels are probably due to deficiencies in the synthesis, processing, transport, or stability of the mRNA coding for the α^Chumphae^ chain rather than to tetramer instability. Although the abnormal chain may be normally synthesized, it is rapidly proteolyzed owing to its polarity and the negatively charged aspartic acid, possibly disturbing the A helix conformation [36].

A notable feature of Hb H-Chumphae is the presence of Hb Bart’s (γ_4_) rather than Hb H (β_4_). A previous case report described a two-week-old Thai boy with Hb H and Hb Bart’s accounting for 1.5% and 29.4% of the total Hb count, respectively [10]. The presence of Hb Bart’s (γ_4_) indicates excess free γ-globin chains not bound to *α-globin* chains, distinctly observed on the chromatogram. Hb H disease typically involves one functional *α-globin* gene, causing a severe imbalance in globin chain synthesis. This imbalance causes excess β-globin chains to precipitate and form β-globin tetramers, clinically identified as Hb H within RBCs. Typically, Hb H disease is characterized by readily detectable Hb H levels in peripheral blood by various analytical techniques. However, Hb H may be absent or reduced during acute hemolytic episodes due to preferential destruction of unstable tetramers. The current patient’s α-globin genotype (α^Chumphae^α/--^SEA^) with normal *β-globin* gene indicates a severe imbalance in globin chain synthesis. Hb H is ideally synthesized in large quantities in adults owing to high *β-globin* gene expression, as adults have more β-globin in RBCs than infants. However, the current patient was experiencing a hemolytic crisis at the time of the investigation. This may have contributed to the failure of HPLC and CE to detect Hb H. Failure to detect Hb H by HPLC may reduce the accuracy of Hb H disease diagnosis. Therefore, molecular testing is essential to improve diagnostic accuracy.

The phenotypic symptom observed in this patient was severe anemia (Hb: 3.7 g/dL and Hct: 17.0%), similar to infants with the same genotype [10]. Co-inheritance of Hb Chumphae and α^0^-thalassemia, typically genotypic Hb H disease, is associated with more pronounced α-globin deficit in non-deletional Hb H disease (--/α^T^α) than in deletional forms (–/-α). Consequently, non-deletional Hb H disease results in more severe clinical symptoms and requires more intensive medical attention than deletional Hb H disease [37].

This study had some limitations. No individuals heterozygous for Hb Chumphae were identified, limiting the understanding of red cell parameters in this population. It is unclear how Hb Chumphae is expressed or whether it can be detected in a simple heterozygous state. However, our data suggest predicted red blood cell profiles similar to those of α^+^-thalassemia carriers, with the Hb variant completely separated from Hb A at low concentrations. Further studies are required to explore the prevalence and clinical impact of Hb Chumphae and other rare Hb variants in diverse populations.

## Conclusion

This study expands the understanding of the hematological profile, genetic background, physicochemical properties, and structural alterations of Hb Chumphae (HBA2:c.32T>A). Particularly, Hb Chumphae (HBA2:c.32T>A) is associated with the α-thalassemia phenotype, with pathogenicity supported by computational predictions and functional analyses. Further, it is associated with severe clinical outcomes when co-inherited with α^0^-thalassemia, highlighting its clinical significance. The migration patterns being similar to those of other Hbs in the Thai population complicate diagnosis. Therefore, molecular-level analysis is essential for accurate detection. New molecular techniques improve diagnostic accuracy.

## Author contributions

P Sihathip: Methodology, Data curation, Writing – original draft. W Srisuwan: Methodology. T Sumphanapai: Writing – original draft, Software. S Satthakarn: Writing – original draft, Validation, Methodology, Formal analysis. T Ngokwongs: Methodology, Data curation. S Panyasai: Conceptualization, Writing – review & editing, Writing – original draft, Validation, Methodology, Formal analysis, Data curation, Funding acquisition. All authors agree to be accountable for all aspects of this study.

## Funding

This work was supported by the Thailand Science Research and Innovation Fund and the University of Phayao, Thailand (grant number 254/2567).

## Conflicts of interest

The authors declare no conflicts of interest.

## Data Availability

All essential data are available in the article. Any additional information is available from the corresponding author upon reasonable request.
